# Implementing the Synchronized Global Switch from Trivalent to Bivalent Oral Polio Vaccines—Lessons Learned From the Global Perspective

**DOI:** 10.1093/infdis/jiw626

**Published:** 2017-07-04

**Authors:** Alejandro Ramirez Gonzalez, Margaret Farrell, Lisa Menning, Julie Garon, Hans Everts, Lee M. Hampton, Samantha B. Dolan, Stephanie Shendale, Sarah Wanyoike, Chantal Laroche Veira, Gaël Maufras du Châtellier, Feyrouz Kurji, Jennifer Rubin, Liliane Boualam, Diana Chang Blanc, Manish Patel

**Affiliations:** 1 World Health Organization, Geneva, Switzerland;; 2 UNICEF Programme Division, New York, New York;; 3 Emory University,; 4 Centers for Disease Control and Prevention, and; 5 Task Force for Global Health, Atlanta, Georgia;; 6 McKing Consulting and; 7 FDK Consulting, Seattle, Washington;; 8 UNICEF, West and Central Africa Regional Office, Dakar, Senegal; and; 9 UNICEF Supply Division, Copenhagen, Denmark

**Keywords:** Polio, eradication, poliovirus, endgame, OPV, oral polio vaccine, IPV, inactivated polio vaccine.

## Abstract

In 2015, the Global Commission for the Certification of Polio Eradication certified the eradication of type 2 wild poliovirus, 1 of 3 wild poliovirus serotypes causing paralytic polio since the beginning of recorded history. This milestone was one of the key criteria prompting the Global Polio Eradication Initiative to begin withdrawal of oral polio vaccines (OPV), beginning with the type 2 component (OPV2), through a globally synchronized initiative in April and May 2016 that called for all OPV using countries and territories to simultaneously switch from use of trivalent OPV (tOPV; containing types 1, 2, and 3 poliovirus) to bivalent OPV (bOPV; containing types 1 and 3 poliovirus), thus withdrawing OPV2. Before the switch, immunization programs globally had been using approximately 2 billion tOPV doses per year to immunize hundreds of millions of children. Thus, the globally synchronized withdrawal of tOPV was an unprecedented achievement in immunization and was part of a crucial strategy for containment of polioviruses. Successful implementation of the switch called for intense global coordination during 2015–2016 on an unprecedented scale among global public health technical agencies and donors, vaccine manufacturers, regulatory agencies, World Health Organization (WHO) and United Nations Children’s Fund (UNICEF) regional offices, and national governments. Priority activities included cessation of tOPV production and shipment, national inventories of tOPV, detailed forecasting of tOPV needs, bOPV licensing, scaling up of bOPV production and procurement, developing national operational switch plans, securing funding, establishing oversight and implementation committees and teams, training logisticians and health workers, fostering advocacy and communications, establishing monitoring and validation structures, and implementing waste management strategies. The WHO received confirmation that, by mid May 2016, all 155 countries and territories that had used OPV in 2015 had successfully withdrawn OPV2 by ceasing use of tOPV in their national immunization programs. This article provides an overview of the global efforts and challenges in successfully implementing this unprecedented global initiative, including (1) coordination and tracking of key global planning milestones, (2) guidance facilitating development of country specific plans, (3) challenges for planning and implementing the switch at the global level, and (4) best practices and lessons learned in meeting aggressive switch timelines. Lessons from this monumental public health achievement by countries and partners will likely be drawn upon when bOPV is withdrawn after polio eradication but also could be relevant for other global health initiatives with similarly complex mandates and accelerated timelines.

Since the World Health Assembly (WHA) resolved to eradicate polio in 1988, polio cases have declined dramatically, from >350 000 cases annually to 37 cases in 2016 [1]. In the past decade, the world has made significant progress toward polio eradication, including the elimination of endemic transmission of polio in all countries worldwide except Afghanistan, Nigeria, and Pakistan. Indeed, as of 23 September 2015, the World Health Organization (WHO) declared that type 2 wild poliovirus had been eradicated, with the last reported case occurring in 1999 [2].

With the eradication of type 2 wild poliovirus, the world is well into the endgame phase of polio eradication, marked by the global introduction of inactivated polio vaccine (IPV) and phased removal of oral polio vaccine, the containment of remaining polioviruses in laboratories and manufacturing facilities, and the transitioning of polio resources to other public health efforts [3, 4]. Although the world is getting closer to eradication, since types 1 and 3 wild polioviruses have not yet been eradicated, use of OPV continues in many countries worldwide. The withdrawal of OPV is taking place in a phased manner, beginning with the removal of the type 2 component of OPV (OPV2), through a global switch from trivalent oral polio vaccine (tOPV), containing live attenuated poliovirus types 1, 2, and 3, to bivalent oral polio vaccine (bOPV), containing poliovirus types 1 and 3. Ultimately, the world must cease using all OPV after the eradication of polioviruses, to avoid the transmission of vaccine-related polioviruses and ensure that polio is eradicated.

Switching from tOPV to bOPV is not without risks [4–10]. In the postswitch era, the primary risk is the reemergence of outbreaks involving type 2 circulating vaccine-derived polioviruses (cVDPV2s) in the context of declining population immunity to type 2 poliovirus following withdrawal of OPV2. To mitigate risks related to the reemergence of type 2 viruses, the WHO Strategic Advisory Group of Experts on Immunization (SAGE) proposed risk mitigation activities that have been described elsewhere [4, 6, 7, 11–17]. In the long run, cessation of OPV2 use by immunization programs worldwide should eliminate the risk for outbreaks of cVDPV2 infection, but in the short run, a prolonged, staggered OPV2 withdrawal would pose a risk for continuous generation of VDPV2s and potential exportation of these viruses to regions or countries with susceptible children born after cessation of OPV2 use. Global synchronization of OPV2 withdrawal within a limited time frame was considered the best approach to minimizing this risk. Scheduling the synchronized cessation of OPV2 use during months when endemic circulation of polioviruses in tropical countries is at its lowest point further reduced the risk of poliovirus type 2 infection outbreaks. The WHA endorsed these SAGE recommendations in May 2015 [18], and all OPV-using countries agreed to switch from tOPV to bOPV during a 2-week time period, from 17 April to 1 May 2016 [19].

While most countries had previous experience replacing one vaccine with another (ie, the global transition from diphtheria, tetanus, and pertussis vaccines to newer combination vaccines that also contained hepatitis B virus and *Haemophilus influenzae* type b antigens), replacement was normally accomplished by depleting existing vaccine stocks and then gradually introducing the new vaccine [20, 21]. One case in which every health facility in a country had simultaneously switched from an existing vaccine to a new vaccine on the same day was the 2010 US switch from 7-valent pneumococcal conjugate vaccine (PCV7) to 13-valent pneumococcal conjugate vaccine (PCV13). For this transition, the manufacturer of both vaccines was controlling and monitoring all cold chain stores that kept PCV7 and PCV13, buying back all PCV7 in existence at the time of the switch, and directing its thousands of sales representatives to monitor stock levels at every health facility using pneumococcal conjugate vaccines, to ensure that there were no stock-outs and that all extra PCV7 was returned [22]. Given this context, the task of synchronizing the switch from tOPV to bOPV so that all vaccine stores and health facilities within a country would switch on the same day and that all countries would switch within a 2-week period, without reimbursement for unused tOPV that was disposed of, was unprecedented. The implementation of the switch was further complicated by uncertainty over whether transmission of VDPV2s would be sufficiently controlled to allow the switch to safely go forward [17], as well as by competing priorities from other global health initiatives (eg, introductions of new vaccines) [23], emergencies, conflicts or natural disasters [24, 25], and large outbreaks of diseases (eg, Ebola and Zika) [26] that could hinder its implementation.

## GLOBALLY SYNCHRONIZED SWITCH FROM TRIVALENT OPV TO BIVALENT OPV—A MONUMENTAL PUBLIC HEALTH ACHIEVEMENT

The Global Polio Eradication Initiative (GPEI) selected the dates for the globally synchronized switch in consultation with World Health Organization (WHO) and United Nations Children’s Fund (UNICEF) regional offices. A 2-week global window was selected, rather than a single fixed date, to provide programmatic flexibility, allowing countries to adjust their processes and to set more-feasible and more-realistic targets. SAGE unanimously endorsed the recommended dates of the global switch window in its meeting during October 2015 [19].

Before the switch, manufacturers reduced and ultimately stopped production of tOPV, resulting in limited availability of tOPV supply leading up to the switch and its unavailability after the switch. Countries were asked to select a date within the 2-week global switch window, after which they would cease all use of tOPV and, within 2 weeks thereafter, validate that tOPV was no longer being administered. After discussions with WHO regional offices, Indonesia, Rwanda, and Ghana selected switch dates a few days earlier than the global window because of logistical considerations.

All countries were encouraged to begin switch planning during the first half of 2015 and to finalize a budgeted national switch plan by September 2015 [27]. Of the 155 countries and territories using OPV in early 2015, 118 (76%) had met this milestone by September, and 147 had met it by the end of 2015. Of the 155 countries and territories, 98% (including Belarus, Malaysia, Poland, Tokelau, and Tuvalu, which changed to an IPV-only schedule) reported ceasing use of tOPV by 1 May, and 100% reported stopping use of tOPV by 12 May 2016 [28, 29]. By the time of the 69th Meeting of the WHA, on 26 May 2016, independent monitoring of cold chain facilities had begun in all countries and territories participating in the switch; of the 155 countries and territories using OPV in early 2015, 147 (95%) had provided WHO with an official report validating the country to be free of tOPV [36]. Seven remaining countries provided the official report to the WHO by mid-September 2016. Although ongoing surveillance for remaining tOPV is crucial, the globally synchronized cessation of tOPV use was altogether an unprecedented and successful public health achievement.

## OVERVIEW OF THE GLOBAL SWITCH GUIDANCE

The global guiding principles for planning the switch focused on maintaining adequate and uninterrupted supply of tOPV up until the time of the switch while avoiding significant excess tOPV stocks after the switch [27]. The challenge for each country was to find this optimal balance. Stock-outs of tOPV before the switch would leave children unimmunized against polio, whereas residual stocks of tOPV could increase the risk for tOPV use after the switch and increase costs associated with the destruction of vaccine doses. Therefore, accurate forecasting, careful procurement planning, close inventory management, and regular monitoring of stock levels were identified as critical actions for countries to minimize wastage of vaccine after the switch.

The switch in its entirety, at the global, regional, and country levels, was an enormous task. Global guidance envisaged the switch activities at the country level to be segregated into 4 phases (Table A1): plan, prepare, implement, and validate [27]. Breaking the stages of the switch into smaller, manageable activities supported the assertion that the switch was feasible. This, combined with a clear delineation of roles and responsibilities for each of the conceived activities at the country, region, and global levels, provided further reassurance of the feasibility of meeting the desired milestones of the switch. The guidance detailed a set of discrete activities and timelines within each of the 4 phases that were likely to be applicable to most of the countries, including 2 key initial milestones to be completed by September 2015: a national tOPV inventory and a budgeted national switch plan. Countries were advised to adapt the guidance to meet their specific needs and, in consultation with stakeholders and partners, develop written national switch plans outlining specific activities that would need to be completed to ensure a successful switch within the country (Table 1). Furthermore, countries and decision-makers were encouraged to conduct a series of activities to facilitate the preparation and implementation of the switch (Table 2).

**Table 1.** Guidance for Development of a National Plan to Switch From Trivalent Oral Polio Vaccine (tOPV) to Bivalent OPV (bOPV)

Guideline Section, Key Component	Comments
Executive summary	
Summary of the switch plan activities	…
Date selected for the national switch day	…
Overview of national coordination mechanism	…
Capacity to implement the switch (eg, financial needs and resources)	…
List of preparatory activities, including plans for tOPV inventory	…
tOPV disposal and validation strategy	…
Key risks and mitigating strategies: supply, logistics, and validation	…
Key milestones and activities	…
Management and operational oversight of switch (national coordination mechanisms)	
Organizational chart with roles and responsibilities	ICC or national switch committee, subnational switch committees, switch support teams
Information flow	Description of who informs whom and with what frequency
Budget for switch activities	…
Work plan and timeline	…
Validation committee	
Roles and responsibilities	…
Validation and reporting process	…
Situation analysis	
Supply and distribution process for OPV	Covers activities in the public and private sectors
Licensing and regulatory approvals needed for bOPV	…
Capacity of existing medical waste management system	…
Stock of tOPV and bOPV to date	…
Preparation	
Switch support	Available budget, composition of switch support team, and communications materials and dissemination
Supply assessment	National inventory of tOPV, plan for tOPV procurement, and plan for bOPV procurement, storage, and distribution
Logistics	Plan for healthcare worker training and supervision, delivering bOPV to service points, and tOPV recall and disposal
Monitoring	Process monitoring (ie, assessing switch activities/milestones) and outcome monitoring (ie, collecting monitoring data and validating tOPV removal)

Abbreviation: ICC, interagency coordination committee.

**Table 2.** Overview of the Switch-Specific Activities That Countries Had to Consider Before the Switch From Trivalent Oral Polio Vaccine (tOPV) to Bivalent OPV (bOPV)

Activity	Description
Select a national switch date	Select 1 day during the switch window when tOPV would be removed from all facilities, sent for proper disposal, and replaced with bOPV.
Establish management structures	Assemble switch coordination committees at national and subnational levels, preferably by mid-2015, using existing in-country structures for coordinating polio eradication or immunization activities, such as an ICC. These committees were responsible for developing switch plans and providing implementation oversight.
Conduct tOPV inventories	Conduct at least 2 national inventories, with the first detailed inventory completed by September 2015.
Map and coordinate bOPV vaccine registration	Perform these activities with national regulatory authorities and manufacturers before the switch.
Develop a switch plan	Finalize a written national switch plan by September 2015 by using the recommended template, leaving approximately 10 months to prepare and implement activities.
Prepare for the switch	Operationalize national switch plans in preparation for switch day. Priority activities included training health workers and logisticians, distributing bOPV to periphery stores, and withdrawing and disposing tOPV according to the timelines outlined in their plan. Countries should have hired or designated staff (ie, switch support teams) to prepare and implement the switch plan.
Implement the switch	Stop using and destroy the remaining stocks of tOPV after the designated national switch day, between 17 April and 1 May 2016.
Validate absence of tOPV	Validate that facilities across the country were free of tOPV during the 2 weeks following the switch date, using WHO-provided guidance on monitoring and validation.
Complete national validation	Delegate authority to an independent body (ie, a national switch validation committee) to review monitoring data and assess whether the country was free of tOPV within 2 weeks of the national switch date.

Abbreviations: ICC, interagency coordination committee; WHO, World Health Organization.

## ACCELERATED GLOBAL HEALTH INITIATIVES NEED CLEAR MESSAGING AND ADEQUATE LEAD TIMES

**Figure 1.**
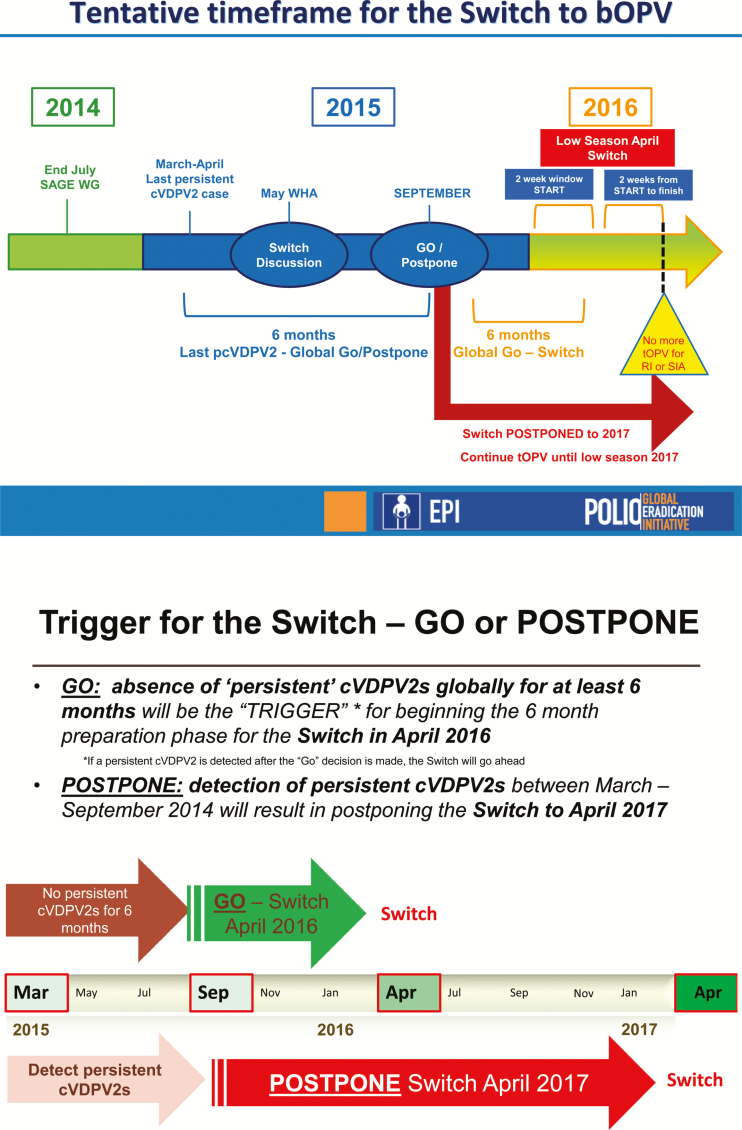
Initial messaging on tentative time frame for the globally synchronized switch from trivalent oral polio vaccine (tOPV), August 2014. Abbreviations: cVDPV2,circulating vaccine-derived poliovirus type 2; pcVDPV2, persistent circulating vaccine-derived poliovirus type 2; RI, routine immunization; SAGE WG, Strategic Advisory Group of Experts on Immunization Working Group; SIA, supplementary immunization activities; WHA, World Health Assembly.

Providing sufficient advance notice of expectations and timelines to countries, partners, and manufacturers was one of the key factors for the successful switch. At the outset, the GPEI decision on the timing of the switch (Figure 1) was challenging to communicate, difficult to understand, and perceived as infeasible for meeting logistical requirements of implementing the switch. The April 2016 switch date was contingent on a series of epidemiologic and operational readiness criteria. These criteria included the introduction of at least 1 dose of IPV in all countries, the licensure of bOPV for use in routine immunization in all countries, the enhancement of environmental (sewage) surveillance for polioviruses, the creation of a monovalent OPV2 (mOPV2) stockpile for use in any postswitch VDPV2 infection outbreaks, the containment of remaining type 2 polioviruses, and the verification of the eradication of wild type 2 polioviruses [30]. Moreover, all countries had to be free of persistent cVDPV2s, which were cVDPV2s of the same genetic lineages that had been in circulation for ≥6 months [31, 32]. The detection of persistent cVDPV2s was thought to represent a polio eradication program failure since such cVDPV2s had been identified but not eliminated. Such persistent cVDPV2s would also indicate that localized immunity to poliovirus type 2 would be insufficient to justify risking OPV2 withdrawal, which would further reduce immunity and risk the spread of an outbreak. As such, the final so-called go-versus-postpone decision for announcing the switch could not be made until October 2015, well after country planning needed to be initiated. No detection of persistent cVDPV2s during the 6 months before September 2015 would trigger the switch in April 2016, providing countries with 6 months of planning time, whereas any detection during that period would delay the switch until at least April 2017. The ramifications of detecting persistent cVDPV2s between October 2015 and April 2016 were unclear.

To balance the need for epidemiologic conditions conducive to a synchronized global switch and the preparatory time needed by countries and manufacturers, GPEI initially assumed that 6 months of preparation would be sufficient after the global readiness criteria were met. However, after further consultations with countries, partners, and stakeholders, GPEI recognized that at least 1 year of advance planning of the switch was necessary. Activities such as vaccine procurement, inventories, securing of budget, bOPV licensing, advocacy, training, and establishing validation processes warranted significant advanced consideration and adequate lead time.

Therefore, in April 2015, SAGE concluded that progress toward elimination of persistent cVDPV2s was on track and that countries should plan firmly for April 2016 as the designated date for withdrawal of OPV2, providing 12 months of planning time for the synchronized switch [33]. During the 68th WHA, in May 2015, all countries endorsed the proposed timelines and agreed to conduct the global switch in April 2016 [18]. Later, during its meeting in October 2015, SAGE indicated that it would only consider delaying OPV2 withdrawal if the assessed risk of continued cVDPV2 transmission was high [19]. These messages from SAGE were crucial for persuading countries to commit to planning for the switch. In October 2015, after reviewing the progress toward elimination of cVDPV2s and the readiness criteria, SAGE confirmed that every country should stop using tOPV on a single day of its choice between 17 April and 1 May 2016 and remove all stocks of tOPV from service delivery points within 2 weeks of that day.

## LAUNCHING THE SWITCH PLANNING—EARLY OBSTACLES

**Figure 2.**
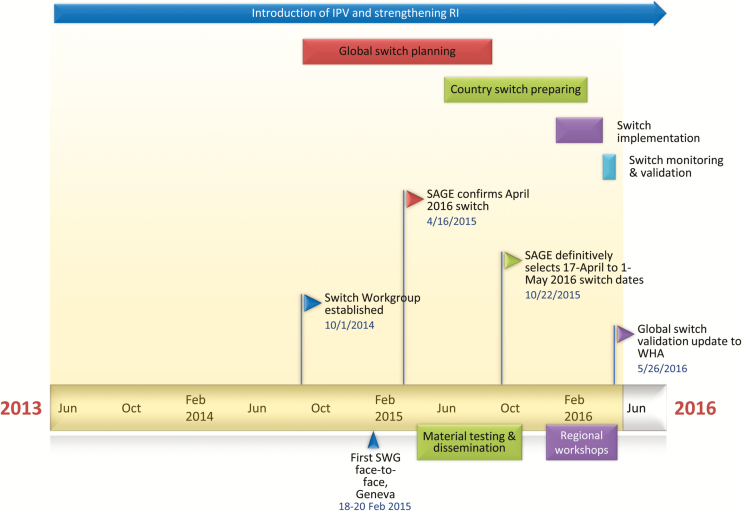
Overview of the switch planning timeline and key milestones. Abbreviations: SAGE, Strategic Advisory Group of Experts on Immunization; SWG, Switch Work Group; WHA,World Health Assembly.

The Immunization Systems Management Group (IMG) was the component of the GPEI tasked with overseeing the introduction of IPV, the switch from tOPV to bOPV, and GPEI’s efforts to strengthen routine immunization. The workload and focus of the IMG shifted during 2013–2016, from IPV introduction to the switch, as the world progressed toward meeting the readiness criteria for the switch. Given the enormity of the task and the accelerated time scale, the IMG focused its efforts during the initial 12–18 months on IPV introduction and strengthening routine immunization services (Figure 2) [34]. Discussions on how to operationalize the global synchronized switch effectively did not begin until mid-2014, followed by the establishment of an official Switch Implementation Working Group (SWG).

Some of the early challenges with developing a work plan, defining roles, and agreeing on the basics of the global strategy were related to the inherent complexities of the task (ie, evolving/variable timelines that were dependent on epidemiologic considerations and the fact that the switch was uncharted territory). Nevertheless, following the first SWG meeting, which occurred in Geneva during February 2015, the working group gained momentum, and the plans for progressing became more clear and consolidated. Moreover, as GPEI progressed toward fulfilling the readiness criteria for the switch, the need to initiate switch planning and better define activities became more pressing [35]. Having a more concrete set of activities and timelines helped during the initial discussions with immunization staff at global, regional, and country levels, many of whom had indicated a general lack of confidence with regard to the idealistic expectations of successful synchronization of the global switch. The synchronized switch was perceived to be a new global health endeavor consisting of unfamiliar activities, with vague definition around the specific tasks (many of which are country specific) that needed completion. These unknowns fostered initial hesitation particularly with respect to the global synchronization component and may have contributed to the inertia in initiating switch-related work globally.

While the switch was ultimately fully accomplished, many of the initial challenges could have potentially jeopardized successful implementation of the switch and, hence, increased the risk for cVDPV2 outbreaks. Specifically, the slow start in initiating the SWG led to inefficiencies and confused messaging that could have been avoided had the necessary human resources been committed at the global level earlier. Nevertheless, the SWG played a critical role in defining milestones, establishing a clear policy and overarching operational objectives and setting firm timelines necessary to achieve the switch.

## COMPLEX TASKS NEED SIMPLE SOLUTIONS

In all, a globally synchronized switch with 12 months of planning seemed an insurmountable task for some partners. However, in identifying the smaller individual components and tasks of the switch, the SWG recognized that the switch was no different from other immunization activities, including planning, inventory and stock management, regulatory approval, establishment of technical advisory and management groups, training, communications and advocacy, monitoring and validation, and waste management [27]. Also, the SWG translated the overall vision for the switch into specifically achievable operational components, delineating the core activities and specifying timelines for achieving each objective. This parceling of the switch into smaller, defined tasks provided the necessary initial motivation for launching detailed country-specific plans and activities. Moreover, recognition that the switch actually was a set of activities familiar to immunization staff was instrumental in boosting confidence among GPEI partners and OPV-using countries and territories in the feasibility of achieving the switch objectives.

## DEVELOPING A GLOBAL SWITCH WORK PLAN FACILITATED PROGRESS

Working from the adage that countries are more alike than different, the SWG focused on developing guidance for core activities that would be relevant and necessary to meet the ultimate goal and that countries could adapt as needed. The SWG developed a comprehensive work plan with priority activities that warranted action at global, regional, and country levels. Partner agreement on these core activities, including assigning roles and responsibilities, was crucial for clarifying global roles and for advancing regional- and country-level planning with regard to the switch. The SWG realized that the work plan needed to be simple and standardized to the greatest extent possible, identifying core activities within each of the key components of the switch, including decision making, management structure, stock management, training, communications, monitoring, disposal, and validation. Keeping the documents simple and standardized allowed regions and countries to advance the development of their own switch plans that were tailored to meet country-specific needs. This template motivated global, regional, and country partners to identify key activities specific to them, enabling each partner to conceive their individual roles and responsibilities.

As the switch neared, detailed guidance on logistic protocols, budget templates, monitoring and validation frameworks, and health worker training modules were drafted and disseminated for regional and country adaptation and use, if needed [27]. In this process, some of the initial messages evolved or gained more clarity and depth. Standardizing guidance on switch activities that were likely to be similar across countries provided countries with starting templates for launching their own switch planning process. Standardizing guidance also facilitated a rapid and clear understanding of the key activities that global, regional, and country partners had to undertake to accomplish a globally synchronized switch within 12 months. Although not all aspects of the standardized guidance and tools were applicable to all countries, most of the materials were adapted by regions and further by countries themselves to facilitate local workshops and dissemination.

## AVOID PARALYSIS BY ANALYSIS

An important milestone for the SWG was the recognition that certain activities and deliberations could occur in parallel to ongoing communication with countries and global partners. For example, guidance on country monitoring and validation of the switch outcomes was a complicated and debated topic in the SWG because of the inherent complexities of developing normative guidance for validating the switch in the 155 countries and territories that used OPV in 2015 [36]. As such, the SWG sought guidance from the SAGE working group on polio. In parallel to this process, the SWG continued moving forward with regional and country workshops to ensure country preparedness for the switch. However, messaging on core components of switch planning activities such as monitoring and validation was incomplete during the initial regional workshops. Even though countries would have preferred standardized guidance on all the components of the switch, waiting for this level of detail to be available before providing any detailed guidance to countries on the switch would have risked serious delays in promoting country planning. Finding the fine balance between consistent, complete messaging and stalled progress was one of the crucial successes of the switch.

## ADAPTABILITY

A consistent principle among members of the SWG and countries was that of adaptability. The switch was an evolving activity that was defined gradually as the various activities were accomplished by partners and countries worldwide. For example, practical guidance on appropriate disposal of tOPV emerged as a pressing country need. This was a complex topic on which preexisting normative guidance and subject matter expertise was lacking [37]. Comprehensive and practical guidance on the management of tOPV waste went through several iterations as SWG members learned more about the characteristics and implications of different disposal options, consulted with experts on pharmaceutical waste management, and received country-specific feedback. Waste management guidance was ultimately developed and shared with countries; however, some countries already had developed plans in the interim that were difficult to alter. Thus, lessons learned by the SWG were to comprehensively consider all important areas of work early in the process of planning, develop a timeline for rapidly developing and finalizing draft guidance, adapt guidance as new information becomes available and new needs become apparent, and accept that countries may develop customized solutions to common problems. Ongoing modifications of guidance may need to occur in parallel to disseminating draft guidance, and effectively highlighting areas of evolving guidance and their timelines may avoid confusion and foster a sense of flexibility. The adaptability among partners and countries throughout this evolution and tailoring was an important asset to switch success.

## FIELD-TESTING MATERIALS: SWITCH DRY RUNS

Another enabling factor of the switch’s success was the field-testing of switch activities, guidance, and tools in a diverse range of settings worldwide, which provided some key early lessons (Table 3). These week-long exercises were conducted after the first round of global switch guidance was developed, beginning with a dry run in 2 large states of northern India and continuing with similar exercises in Tanzania, Mongolia, and Cameroon. These dry runs used agendas and materials that were initially developed and tested in a workshop and webinars for global consultants. Dry runs were essentially country-level switch planning workshops that involved a diverse set of participants, such as national decision makers, government and private health workers, cold-chain staff, polio workers, communications staff, regulatory agencies, nongovernmental organizations, civil-society organizations, and multilateral agencies [38]. This broad representation reinforced the contention that the success of the switch was contingent on meeting responsibilities that were shared across multisectoral partners. The dry runs involved meetings with multilevel participants (from the national level to the district level), along with field visits to local facilities to provide contextual information and perspective from key field personnel. The most important message to emerge from the dry-runs was that the switch was perceived as feasible. A clear understanding of the rationale for the switch’s timelines and synchronization motivated staff to develop frameworks for the national operational plans, and many participants enthusiastically provided specific ideas to meet switch objectives.

**Table 3.** Key Lessons From the Field Exercises, or Dry-Runs, to Simulate the Switch From Trivalent Oral Polio Vaccine (OPV) to Bivalent OPV

Provision of motivating principles and flexible guidelines fostered engagement, creativity, and ownership among national participants. Some activities needed rigid timelines (eg, switch dates and advance stock inventories), but identifying the process (eg, how to conduct the inventory) needed to be country driven. Representation from national and subnational technical and logistical experts improved cross-fertilization of ideas within the country and provided important feedback for global guidance. While extensive guidance was prepared to address the many components of the switch, document overload, particularly during the first country sensitization and planning missions, could be counterproductive. For example, a detailed budgeting tool that did not consider country context was deemed impractical by country staff for initial workshops. Most important, staff communicated that challenges with the switch were no different from daily in-country challenges facing routine immunization programs. Participants consistently conveyed an optimistic message that the switch was nothing more than what they do on a daily basis.

## EFFECTIVE DISSEMINATION OF SWITCH GUIDANCE MATERIALS

To raise global awareness and confidence on the feasibility of the switch, technical guidance and communication materials on the switch were rapidly disseminated through various official and unofficial channels. Working groups of the IMG routinely updated details on the switch on the WHO website. [27]. Partner webinars were deemed to be an effective means of transmitting switch guidance broadly, providing switch experts with a platform to field-test complex materials, simplify messages, and identify gaps before broader dissemination to countries and implementing agencies. IMG members also leveraged regional and country meetings of EPI managers, technical advisory groups, polio certification committees, and scientific communities and organizations to advance the broad dissemination of switch guidance and advocacy. However, perhaps the most important events for ensuring country input, buy-in, and engagement were switch-specific consultant trainings and regional workshops for country representatives.

## SWITCH AMBASSADORS—CONSULTANT AND COUNTRY WORKSHOPS

In May 2015, the SWG coordinated a global switch training workshop for consultants and regional office focal points from all of the WHO and UNICEF regions. This workshop was instrumental for vetting the initial global switch guidance and was a unique opportunity to raise switch awareness on detailed activities necessary for implementation, to seek input on materials developed to date, and to provide a platform for voicing country and region needs. The workshop attendees became switch ambassadors and later provided invaluable guidance to countries through direct support in country planning and implementation or through other activities (eg, leading and facilitating regional country workshops and dry runs). The materials vetted and revised at this workshop, including the agenda, were modified and replicated throughout the world in numerous regional workshops, webinars, and dry runs and provided the foundation for developing country switch plans. These workshops, webinars, and dry runs were the backbone of the global planning and implementation of the synchronized switch.

## NATIONAL AND REGIONAL OWNERSHIP—A CRITICAL FACTOR FOR SWITCH SUCCESS

Early engagement with the regional offices by the SWG was critical to the success of the switch. Developing a core set of guidance and tools reflecting the switch strategy before engaging the regions and countries provided a useful frame of reference and a platform for concrete discussions. Engagement with regional offices provided the necessary input for customization of the guidance and identified approaches to field-testing the materials rapidly during the dry run. Initial consultations through global workshops and calls included all regional offices simultaneously, which provided transfer and exchange of useful ideas across and within the regions. For example, the South-East Asia Regional Office developed a tracking tool for monitoring country-specific switch progress, and the Regional Office for the Americas developed monitoring and validation tools that were then adapted for other regions of the world. Later, each of the regions adopted globally available materials or developed its own materials that catered to the needs of countries in its region. The SWG maintained close communication and collaboration with the regional offices, and it monitored regional progress through frequent conference calls, emails, and consultations, providing additional guidance and support as needed. Overall, ownership of the switch by the regional offices—by monitoring progress, providing country support, and identifying issues warranting global attention—was critical to the success of the globally synchronized switch.

## CONCLUSION

Global health initiatives are known to have an emergent quality, but the switch also had a compressed timeline. The initial approach to global switch planning did not meet the requirements of the task. As switch planning progressed and the switch window approached, the strengths of the IMG percolated throughout the partnership to help address the early shortcomings of the switch planning (Table 4). Overall, the globally synchronized switch was a success, with all countries reporting the cessation of tOPV use close to SAGE’s recommended timelines. The success of the synchronized switch globally was because of flexibility, clear communication, coordination and collaboration, and strong leadership across all levels, including the GPEI partnership. Dissemination of clear, simple messages gave shape to switch activities, provided optimism and confidence, and allowed accelerated progress globally. Communications were modified as needed to attain more depth and to meet the evolving needs of the regions and countries. Activities among the partners, regions, and countries were effectively coordinated to avoid duplication of efforts, to foster exchange of ideas, and to advocate for necessary resources. A true collaboration developed among all global, regional, and country partners that was built on trust, technical strength, and optimism—an infectious can-do spirit—and resulted in successful withdrawal of OPV2 in a synchronized manner from the cold chain worldwide. The global switch from tOPV to bOPV has set an important precedent regarding the kind of synchronized, cooperative international efforts that are possible and upon which future efforts can be built.

**Table 4.** Summary of Key Challenges and Lessons Learned From the Global Planning of the Switch From Trivalent Oral Polio Vaccine (OPV) to Bivalent OPV

Switch Component	Strengths	Weaknesses	Lessons Learned
Overall	Strong partnership, excellent coordination, adaptability, practicality, and commitment to success	Delays in establishing a switch working group, inadequate resources, no clear work plan and competing priorities among partners at the outset, and pessimism about meeting switch timelines	Early senior leadership and guidance, establish clear vision and objectives, establish clear roles and responsibilities, and foster optimism
Policy and timeline	Strong and comprehensive communication of rationale encouraged buy-in, many contingency plans, objective and risk based, and guided by respected advisory group (SAGE and SAGE Working Group)	Complex messaging (eg, go-versus- postpone decision), unclear timelines for OPV2 withdrawal, inconsistent and late guidance on disposal, and lack of operational considerations	Early incorporation of operational feasibility into policies and timelines, and clear and consistent messaging facilitates optimism and motivates partners
Switch Implementation Working Group	Trust and collaboration; coordination; core team of broad skill sets, right size, and good previous working relationships; and shared mission, responsibility, and absence of personal agenda	Delays in establishing work plan, roles, and responsibilities; and lengthy process of reaching consensus challenging for accelerated switch timelines	Early, multiday, face-to-face meeting crucial for advancing work; important to achieve early agreement on the basics of strategy, structure, and roles; and strategic work plan (complex objectives can be achieved if broken into smaller manageable tasks)
Developing guidance and tools	Comprehensive approach, lead agency with multiagency input, consistency in messaging, rapid turnaround, and multilingual translations	Unclear process of finalizing and disseminating, inadequate use of professional copyediting and communication services, excess documents and tools, and complex guidance early in the switch planning	Simple and standardized allows scalability, strategic dissemination fosters motivation and optimism (simple first, then more complex), provide guiding principles and countries will adapt to meet needs, and plan for copyediting and translating
Field testing materials	Broad platforms (webinars, dry- runs, and workshops), innovative approaches, adaptability, and disseminating and testing simultaneously	Potential for confusion with changing messages and materials, resource intensive, and excess documents	Innovative approach to rapid field-testing of materials; provides platform for global staff to interact with field; once rationale clearly explained, logistics became clearer to participants; advance planning is important but adaptability is crucial; and avoiding document overloads to participants for first country sensitization and planning missions (excess documents can cause confusion)
Consultant and country workshops	Real-world input, enabling ambassadors and advocates, surge capacity of support, passive diffusion of messages, and global followed by regional workshops	Inefficient if consultants not used, inadequate pool of skilled consultants, inability to promise assignments, and language restrictions	Advance planning; invest in roster of consultants; hybrid workshops of consultants, regional, and country office staff useful; and replicate/adapt agenda and materials once tested globally

Abbreviations: OPV2, poliovirus type 2 component of oral polio vaccine; SAGE, Strategic Advisory Group of Experts on Immunization.

**Table A1.** Proposed Switch Calendar for Switch Activities Disseminated to Countries in April 2015

Activity, Time	Description
Plan	
By Jun 2015	Establish management structure, establish NSVC, conduct situational analysis, and draft national switch plan (budgeted and finalized by 1 Sep 2015)
Prepare	
May–Sep 2015	Complete detailed tOPV inventory and adjusted tOPV delivery,^a^ secure funding and finalize national switch plan, and develop monitoring plan
Oct–Nov 2015	Complete second tOPV inventory and adjust tOPV orders and/or delivery, order bOPV, develop waste-management protocol, and hire switch support staff
Dec 2015–Jan 2016	Receive last tOPV delivery in country,^b^ redistribute remaining tOPV stock within country as required, prepare training materials and implement communications strategy, and begin bOPV deliveries to country^c^
Feb–Mar 2016	Deliver last 1–2-mo supply of tOPV to periphery (redistribute as needed) and identify switch monitors
Implement	
2–4 wks before switch	Train switch monitors, train health workers, and distribute bOPV to periphery and service points
National switch day	
17 Apr–1 May 2016^d^	Stop use of tOPV, remove tOPV from cold chain, and begin use of bOPV
Validate	
During 2 wks after switch	Validate tOPV disposal at selected sites (switch monitors) and collect and review data and validate switch (NSVC)

Abbreviations: bOPV, bivalent oral polio vaccine; NSVC, national switch validation committee; OPV, oral polio vaccine; tOPV, trivalent oral polio vaccine.

^a^tOPV orders and delivery can vary on the basis of a country’s ordering cycle.

^b^Unless there is a tOPV stock-out.

^c^Could extend to March 2016 because of logistical delays.

^d^The interval for switching was selected by the Strategic Advisory Group of Experts on Immunization in October 2015.

## Notes

***Disclaimer.***   The findings, interpretations, and conclusions expressed in this article are those of the authors and do not necessarily reflect the policies or views of UNICEF, the WHO, the Bill and Melinda Gates Foundation, or the Centers for Disease Control and Prevention.

***Financial support.***  This work was supported by the Bill and Melinda Gates Foundation (grant OPP1095024 to M. M. P., J. G., C. L. V., and S. W. and contracts to F. K. and H. E.).

***Supplement sponsorship.*** This work is part of a supplement coordinated by the Task Force for Global Health with funding provided by The Bill and Melinda Gates Foundation and the Centers for Disease Control and Prevention.

***Potential conflicts of interest.***  All authors: No reported conflicts. All authors have submitted the ICMJE Form for Disclosure of Potential Conflicts of Interest. Conflicts that the editors consider relevant to the content of the manuscript have been disclosed.

## References

[1] Global Polio Eradication Initiative, World Health Organization, Geneva, Switzerland. http://www.polioeradication.org/Dataandmonitoring/Poliothisweek.aspx. Accessed 15 February 2017.

[2] Global Polio Eradication Initiative, World Health Organization, Geneva, Switzerland. http://www.polioeradication.org/mediaroom/newsstories/Global-eradication-of-wild-poliovirus-type-2-declared/tabid/526/news/1289/Default.aspx. Accessed 15 February 2017.

[3] Global Polio Eradication Initiative. Polio Eradication and Endgame Strategic Plan 2013–2018. WHO/POLIO/13.02 http://www.polioeradication.org/Portals/0/Document/Resources/StrategyWork/PEESP_EN_US.pdf 2013 Accessed 24 August 2016.

[4] Patel M, Orenstein W A world free of polio–the final steps. N Engl J Med 2016; 374:501–3. 26863350 10.1056/NEJMp1514467

[5] Duintjer Tebbens RJ, Pallansch MA, Kim JH Oral poliovirus vaccine evolution and insights relevant to modeling the risks of circulating vaccine-derived polioviruses (cVDPVs). Risk Anal 2013; 33:680–702. 23470192 10.1111/risa.12022 PMC7890645

[6] Garon J, Seib K, Orenstein WA Polio endgame: the global switch from tOPV to bOPV. Expert Rev Vaccines 2016; 15:1–16. 26751187 10.1586/14760584.2016.1140041

[7] Patel M, Zipursky S, Orenstein W, Garon J, Zaffran M Polio endgame: the global introduction of inactivated polio vaccine. Expert Rev Vaccines 2015; 14:749–62. 25597843 10.1586/14760584.2015.1001750

[8] Tebbens RJ, Pallansch MA, Kew OM Risks of paralytic disease due to wild or vaccine-derived poliovirus after eradication. Risk Anal 2006; 26:1471–505. 17184393 10.1111/j.1539-6924.2006.00827.x

[9] Thompson KM, Duintjer Tebbens RJ Modeling the dynamics of oral poliovirus vaccine cessation. J Infect Dis 2014; 210:S475–84. 25316870 10.1093/infdis/jit845

[10] Thompson KM, Tebbens RJ Current polio global eradication and control policy options: perspectives from modeling and prerequisites for oral poliovirus vaccine cessation. Expert Rev Vaccines 2012; 11:449–59. 22551030 10.1586/erv.11.195

[11] World Health Organization. Strategic Advisory Group of Experts on immunization (SAGE). Geneva: WHO, 20–22 October 2015 http://www.who.int/immunization/sage/meetings/2015/october/sage_report_oct_2015.pdf?ua=1 Accessed 26 October 2015.

[12] World Health Organization. Meeting of the SAGE Polio Working Group. Geneva: WHO, 7–8 September 2015 http://www.who.int/immunization/sage/meetings/2015/october/2_SAGE_WG_report_draft_Final_clean.pdf?ua=1 Accessed 30 October 2015.

[13] Meeting of the Strategic Advisory Group of Experts on immunization, April 2012—conclusions and recommendations. Wkly Epidemiol Rec 2012; 87:201–16. 24340402

[14] Meeting of the Strategic Advisory Group of Experts on immunization, November 2012—conclusions and recommendations. Wkly Epidemiol Rec 2013; 88:1–16. 23311010

[15] Meeting of the Strategic Advisory Group of Experts on immunization, April 2013—conclusions and recommendations. Wkly Epidemiol Rec 2013; 88:201–6. 23696983

[16] Meeting of the Strategic Advisory Group of Experts on immunization, November 2013—conclusions and recommendations. Wkly Epidemiol Rec 2014; 89:1–20. 24466571

[17] Meeting of the Strategic Advisory Group of Experts on immunization, April 2014—conclusions and recommendations. Wkly Epidemiol Rec 2014; 89:221–36. 24864348

[18] World Health Organization, Sixty-eighth World Health Assembly: Poliomyelitis: Report by the Secretariat http://apps.who.int/gb/ebwha/pdf_files/WHA68/A68_21-en.pdf Accessed 24 August 2016.

[19] Meeting of the Strategic Advisory Group of Experts on immunization, October 2015—conclusions and recommendations. Wkly Epidemiol Rec 2015; 90:681–99. 26685390

[20] Gupta SK, Sosler S, Lahariya C Introduction of haemophilus influenzae type b (Hib) as pentavalent(DPT-HepB-Hib) vaccine in two states of india. Indian Pediatr 2012; 49:707–9. 23024078 10.1007/s13312-012-0151-0

[21] Sadoh AE, Nwaneri DU, Ogboghodo BC, Sadoh WE Effect of introduction of pentavalent vaccine as replacement for diphtheria-tetanus-pertussis and hepatitis B vaccines on vaccination uptake in a health facility in nigeria. Vaccine 2016; 34:2722–8. 27108191 10.1016/j.vaccine.2016.04.026

[22] Centers for Disease C, Prevention. Licensure of a 13-valent pneumococcal conjugate vaccine (PCV13) and recommendations for use among children—advisory committee on immunization practices (ACIP), 2010. MMWR Morb Mortal Wkly Rep 2010; 59:258–61. 20224542

[23] Carole Tevi-Benissan M, Moturi E, Anya BM Contribution of polio eradication initiative to effective new vaccine introduction in africa, 2010–2015. Vaccine 2016; 34:5193–5198 27396517 10.1016/j.vaccine.2016.05.063

[24] Lam E, McCarthy A, Brennan M Vaccine-preventable diseases in humanitarian emergencies among refugee and internally-displaced populations. Hum Vaccin Immunother 2015; 11:2627–36. 26406333 10.1080/21645515.2015.1096457 PMC4685677

[25] Global polio eradication initiative: 10th meeting of the independent monitoring board. Wkly Epidemiol Rec 2014; 89:361–7. 25136721

[26] Ferguson NM, Cucunubá ZM, Dorigatti I Countering the Zika epidemic in Latin America. Science 2016; 353:353–4. 27417493 10.1126/science.aag0219 PMC5475255

[27] World Health Organization. Guidance for implementing the switch http://www.who.int/immunization/diseases/poliomyelitis/endgame_objective2/oral_polio_vaccine/implementation/en/ Accessed 24 August 2016.

[28] World Health Organization: Global switch in oral polio vaccines—situation report http://maps.who.int/OPV_switch/ Accessed 24 August 2016.

[29] Hampton LM, Farrell M, Ramirez-Gonzalez A Cessation of trivalent oral poliovirus vaccine and introduction of inactivated poliovirus vaccine—worldwide, 2016. MMWR Morb Mortal Wkly Rep 2016; 65:934–8. 27606675 10.15585/mmwr.mm6535a3

[30] World Health Organization. Type 2 OPV withdrawal: Update on readiness and preparations for the switch for April 2015 SAGE session http://www.who.int/immunization/sage/meetings/2015/april/4_Switch_Update_Background_Paper_April_SAGE_2015_22mar15HJREV.pdf Accessed 11 September 2016.

[31] World Health Organization. 9th Meeting of the SAGE Polio Working Group. Geneva: WHO, 30–31 July 2014 http://www.who.int/immunization/sage/meetings/2014/october/1_SAGE_Note_for_the_Recordv14July2014.pdf?ua=1 Accessed 25 August 2016.

[32] World Health Organization. OPV cessation. Protocol for a global coordinated switch from trivalent OPV to bivalent OPV Draft version 9 October 2014 http://www.who.int/immunization/sage/meetings/2014/october/2_Switch_Protocol_SAGE_Version_9_Oct14.pdf?ua=1 Accessed 25 August 2016.

[33] Meeting of the Strategic Advisory Group of Experts on immunization, April 2015: conclusions and recommendations. Wkly Epidemiol Rec 2015; 90:261–78. 26027016

[34] Introduction to inactivated polio vaccine and switch from trivalent to bivalent oral poliovirus vaccine worldwide, 2013–2016. Wkly Epidemiol Rec 2015; 90:337–43. 26151981

[35] Meeting of the strategic advisory group of experts on immunization, October 2014—conclusions and recommendations. Wkly Epidemiol Rec 2014; 89:561–76. 25513671

[36] Farrell M Monitoring and Validation of the Global Replacement of tOPV with bOPV, April–May 2016. J Infect Dis 2017; 216 (suppl 1):S193–201. 10.1093/infdis/jiw558 PMC5853513 28838162

[37] Wanyoike S, Ramirez-Gonzalez A, Dolan S Disposing of excess vaccines after the withdrawal of oral polio vaccine. J Infect Dis 2017; 216 (suppl 1):S202–8. 10.1093/infdis/jiw572 PMC5853297 28838168

[38] Global Immunization News: July 2015—Workshop on planning for the switch in oral polio vaccines in Mongolia http://www.who.int/entity/immunization/GIN_July_2015.pdf?ua=1 Accessed 24 August 2016.

